# *Lycium barbarum* Polysaccharides Attenuate Cisplatin-Induced Hair Cell Loss in Rat Cochlear Organotypic Cultures

**DOI:** 10.3390/ijms12128982

**Published:** 2011-12-06

**Authors:** Quan Liu, Yanqing Li, Li Hu, Dehui Wang

**Affiliations:** 1 Department of Otolaryngology, Eye, Ear, Nose and Throat hospital, Shanghai Medical College of Fudan University, Shanghai 200031, China; E-Mails: liuquan0607@yahoo.cn (Q.L.); qqhbc007@126.com (Y.L.); 2 Central Laboratory, Eye, Ear, Nose and Throat hospital, Shanghai Medical College of Fudan University, Shanghai 200031, China; E-Mail: hl318@yahoo.com.cn

**Keywords:** *Lycium barbarum* polysaccharides, cisplatin, hair cells, reactive oxygen species, mitochondria

## Abstract

The aim of the present study was to investigate the effects of *Lycium barbarum* polysaccharides (LBP) on cisplatin-induced hair cell damage in the organ of Corti explant. The neonatal (P2–3) rat organ of Corti explant was exposed to cisplatin (20 μM; 48 h) with or without LBP pretreatment (150 and 600 μg/mL; 24 h). Hair cell loss was indicated by FITC-labeled phalloidin staining. The level of reactive oxygen species (ROS) and alteration of mitochondrial membrane potential (ΔΨ_m_) in hair cells were analyzed using fluorescent probes 2′,7′-dichlorofluorescein diacetate and JC-1, respectively. The results showed that LBP significantly attenuated hair cell loss (*p* < 0.01). Hair cells pretreated with LBP showed significant reduction in ROS production and the decline of ΔΨ_m_ compared with cisplatin alone group (*p* < 0.01), indicating the protective effect of LBP on cisplatin-induced hair cell loss. Taken together, these results indicate that LBP was effective in attenuating cisplatin-induced hair cell loss by reducing the production of ROS and maintaining mitochondrial ΔΨ_m_.

## 1. Introduction

Cisplatin, an anti-neoplastic agent, has been widely used to treat a broad spectrum of malignant tumors; however, the administration of cisplatin is limited by its side effects, including ototoxicity and nephrotoxicity [1–3]. Increased documentation demonstrated that the generation of reactive oxygen species (ROS), which interferes with the antioxidant defense systems of the cochlea, played an important role in the pathophysiology of cisplatin-induced ototoxicity [4,5]. In the inner ear, the increased ROS caused by cisplatin can induce apoptosis in the cochlear hair cells as well as in the neurons of the spiral ganglion. Some *in vitro* and *in vivo* studies reported the protective effect of antioxidants against cisplatin-induced hair cell death [6,7].

*Lycium barbarum*, a traditional Chinese herbal medicine, which is widely consumed by oriental people, has exhibited anti-cancer and immuno-enhancing activities [8–10]. Recently, *L. barbarum* has been reported to have protective effect against oxidative damage [11–13]. Polysaccharides are the main components isolated from *L. barbarum*. Cheng *et al*. [14] reported that *L. barbarum* polysaccharides (LBP) significantly ameliorated liver injury, prevented the progression of alcohol-induced fatty liver and improved the antioxidant functions in liver. Lin *et al*. [15] indicated that at high concentration, most polysaccharide fractions were effective in scavenging superoxide anion, 2,2-diphenyl-1-picrylhydroxyl as well as hydroxyl radicals. In animal models, some studies suggested that the administration of antioxidants, including cisplatin, protects against hearing loss caused by noise and drugs [16,17].

In our previous study LBP attenuated mitochondrial swelling in spiral ganglion cells in rats treated with cisplatin. Given its antioxidant property, it is conceivable that LBP may be effective in preventing cisplatin-induced hair cell damage. Therefore, this study was aimed to investigate the effects of LBP on cisplatin-induced hair cell damage in the organ of Corti explant.

## 2. Results and Discussion

### 2.1. LBP Protection Against Cisplatin-Induced Hair Cell Death

**Figure 1:**
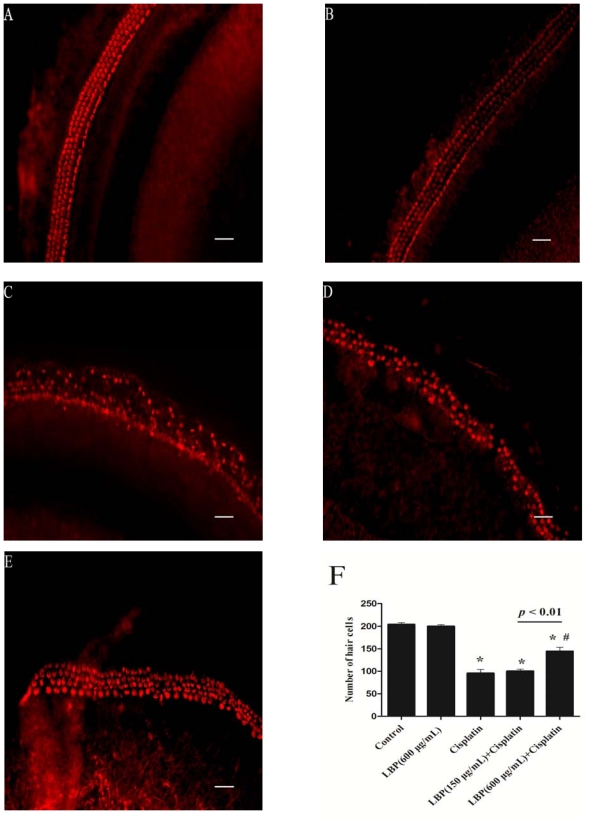
Effects of L. barbarum polysaccharides (LBP) on the cisplatin-induced hair cell loss (mean ± SD). The basal turn of cochlea was used to investigate the effect of LBP on cisplatin induced hair cell loss. * *p* < 0.01 compared with the control group; ^#^ *p* < 0.01 compared with the cisplatin alone group. Scale bar, 20 μm.

To assess the toxicity of cisplatin on hair cells, the organ of Corti explant was exposed to the cisplatin (20 μM) for 48 h. The density of hair cells was analyzed. Figure 1A shows the orderly arrangement of inner hair cells (IHCs) and outer hair cells (OHCs) in the control group, with the arrangement of OHC stereocilia in a V-shaped pattern. Our previous experiment demonstrated that exposure to LBP (600 μg/mL) for 72 h had no damage on hair cells (Figure 1B). Cisplatin exposure for 48 h resulted in substantial loss of OHCs and IHCs compared with the control group (*p* < 0.01; Figure 1C). LBP pretreatment (150 and 600 μg/mL) significantly attenuated hair cell loss in comparison to cisplatin alone group (*p* < 0.01; Figures 1D,E). The number of hair cells shown in Figure 1F suggests that LBP can protect against cisplatin-induced hair cell loss in a dose-dependent manner.

### 2.2. LBP Reduced the Generation of ROS in Hair Cells

**Figure 2:**
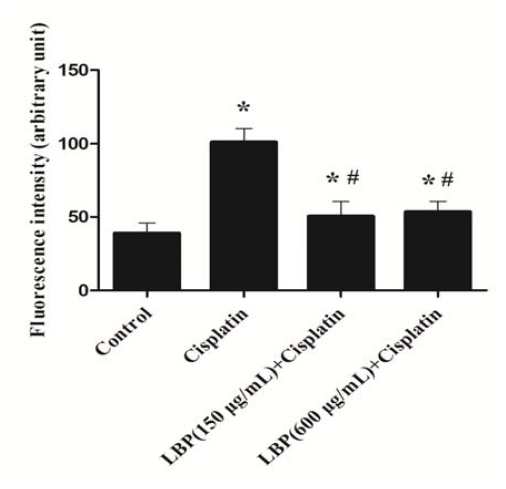
Effects of LBP on the generation of reactive oxygen species (ROS) in hair cells (mean ± SD). * *p* < 0.01 compared with the control group; ^#^ *p* < 0.01 compared with the cisplatin alone group.

The generation of ROS plays a pivotal role in the pathophysiology of cisplatin-induced hearing loss [4,5]. Mitochondria are the important sources for ROS in cisplatin-mediated auditory cell damage [18,19]. Mitochondrial ROS burst is an early upstream apoptotic signal. Oxidative damage to mitochondria causes the impairment of mitochondrial function and subsequently leads to cell death via apoptosis and necrosis [20–22]. Therefore, we investigated the level of ROS in organ of Corti explant using DCFH-DA. Figure 2 shows the alteration of fluorescent intensity in organ of Corti explant 48 h after cisplatin addition. Exposure to cisplatin (20 μM) for 6 h induced a detectable increase of fluorescent intensity for ROS in hair cells (data not shown). Cisplatin exposure for 48 h caused more than a two-fold increase in fluorescent intensity for ROS production in hair cells (*p* < 0.01). A significantly decreased level of fluorescence intensity was discovered in organ of Corti explant pretreated with LBP compared with the cisplatin alone group (*p* < 0.01). The results indicate that LBP can reduce generation of ROS in organ of Corti treated with cisplatin. Our results further confirm that LBP has a protective effect against oxidative damage.

### 2.3. Effects of LBP on Mitochondrial ΔΨ_m_

**Figure 3:**
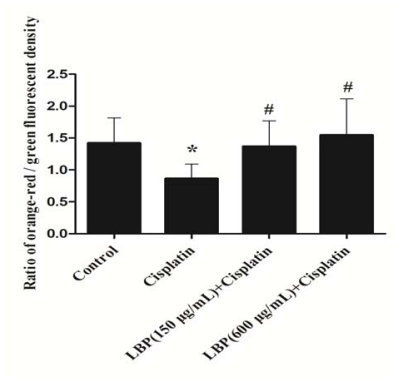
Effects of LBP on mitochondrial ΔΨ_m_ in hair cells (mean ± SD). * *p* < 0.01 compared with the control group. ^#^ *p* < 0.01 compared with the cisplatin alone group.

An enhanced ROS will cause a decrease of mitochondrial membrane potential, which alters cellular energy production and starts mechanisms for the execution of apoptosis [23]. In our study, we found that cisplatin caused mitochondrial ΔΨ_m_ decline in hair cells and that the decline of ΔΨ_m_ can be attenuated by LBP (*p* < 0.01). As shown in Figure 3, the ratio of orange-red/green fluorescent density in cisplatin alone group declined by 43.5% in comparison to the control group and no significant difference in ΔΨ_m_ was found between control group and LBP pretreated groups. The results demonstrate that LBP can inhibit the decline of ΔΨ_m_ in hair cells treated with cisplatin. Our results indicate that LBP protects against cisplatin-induced hair cell damage by maintaining the ΔΨ_m_ in hair cells.

### 2.4. Discussion

The application of cisplatin resulted in progressive and irreversible hearing loss, which is the main limiting factor of the cisplatin dosage in current clinical therapeutic strategies. Ototoxicity is a dose-limiting side effect of chemotherapeutic treatment with cisplatin. Cisplatin-induced ototoxicity is initiated by its uptake into the hair cells and neurons. ROS generation in cisplatin-treated hair cells was closely correlated with its ototoxicity. An increased ROS will cause a decrease of mitochondrial membrane potential, which alters cellular energy production and starts mechanisms for the execution of apoptosis [20,23]. Our results showed that exposure to cisplatin (20 μM) for 6 h increased the generation of ROS in hair cells and about a two-fold increase in ROS production 48 h after cisplatin exposure.

Many antioxidants have been used to prevent cisplatin-induced hearing loss. d-methionine was reported to protect cochlear antioxidant enzyme levels from cisplatin-induced decrements [24]. Systemic administration of d-methionine was proved to have a potential oto-protective role [25]. Sodium thiosulfate has also been described as a protective agent against cisplatin toxicity. Wang *et al*. reported that local application of sodium thiosulfate into the cochleae of guinea pigs prevented cisplatin-induced hearing loss and that a minimal loss of outer hair cells in the organ of Corti was found, indicating the protective effect of sodium thiosulfate against cisplatin toxicity [26]. Neuwelt *et al*. reported that sodium thiosulfate was significantly protective against cisplatin-induced ototoxicity 4 h after cisplatin administration [27].

Increasing evidence indicates that LBP has protective effect against oxidative damage [11–15]. Previous studies have demonstrated that LBP possessed biological activities including anti-aging, anti-tumor, and immune-stimulatory [8,9,28]. To the best of our knowledge, there are only a few studies concerning the effect of LBP on the cisplatin ototoxicity. Our current findings showed that LBP attenuated hair cell loss induced by cisplatin. To investigate the underlying mechanism of the protective effect, we further analyzed the ROS generation in hair cells treated with cisplatin with or without LBP. Our findings indicated that LBP reduced cisplatin-induced intracellular ROS in hair cells treated with cisplatin.

Previous studies demonstrated that epicatechin extracted from the green tea significantly inhibited cisplatin-induced intracellular ROS generation in a cochlear organ of Corti-derived cell line, HEI-OC1. Also, epicatechin treatment prevented cisplatin-induced reduction in ΔΨ_m_ by 40% [29]. To investigate if the LBP can maintain the stability of ΔΨ_m_ in hair cells, we monitored the alterations of ΔΨ_m_ in hair cells treated with cisplatin, with or without LBP. Our results revealed that LBP inhibited the decline of ΔΨ_m_ in hair cells treated with cisplatin, suggesting that LBP played an important role in maintaining the stability of ΔΨm in hair cells.

Our findings indicated the protective effect of LBP against cisplatin ototoxicity. The limitation in the present study, however, was that the protective effect of LBP against cisplatin ototoxicity in organotypic culture may not occur in the *in vivo* model. So, further research needs to be done in animal models to assess the protective effect of LPB.

## 3. Experimental Section

### 3.1. LBP Extract Processing

The dried wolfberries (700 g) were grounded to fine powder, extracted with pure water in reflux for 3 times (800 mL, 700 mL, 700mL) and then concentrated to a volume at 350 mL under vacuum. The concentrated extract was precipitated using 95% ethanol. After centrifugation and several rinses with absolute ethanol, the resulting precipitate was extracted with 6 times volume of 95% ethanol and centrifuged; then the precipitate was dissolved with water and deproteinized. The prepared solution was dialyzed against running distilled water for 24 h. After centrifugation with absolute ethanol, the resulted precipitate was vacuum-dried at 40 °C to yield a brown powder Wolfberry extract-LBP (1.8 g) [11]. The content of LBP was determined as 82.1% by phenol-sulfuric acid method.

### 3.2. Animals and Dissection

Newborn Sprague-Dawley rats (P2–3) were used to prepare cochlear organotypic cultures. All experimental procedures in this study were conducted according to current institutional guidelines for laboratory animal care.

The dissection procedure is similar to that described by Zhang *et al*. [30]. After decapitation, the heads were cleaned with 75% ethanol. The scalp was removed and the skull was transected along mid-sagittal plane. The brain was removed to expose the posterior fossa. The temporal bones were freed from the posterior hemi-skulls and transferred into Petri dishes containing 4 °C PBS. The following procedures were performed on ice. Under a stereomicroscope, the tympanic membrane and annulus were laterally peeled away and the surrounding cartilages were removed to expose the cochlear capsule. The cochlear capsule was carefully removed away. The stria vascularis and spiral ligament were stripped away from the base to the apex, and the organ of Corti was separated away from the modiolus. Our previous experiments in adult rats indicated that the cisplatin caused hearing loss at frequencies of 8, 16, 24 and 32 kHz. So, in the present study, the basal turn of cochlea was used to investigate the effect of LBP on cisplatin induced hair cells loss.

### 3.3. Organ of Corti Culture

A drop (50 μL) of cool, DF12 + 10% FBS was placed in the center of a 24 × 24 mm glass coverslip precoated with 10 μg/mL poly-l-lysine (PLL, MW 70,000–150,000, Sigma-Aldrich, St. Louis, MO, USA) and placed in a 35 mm diameter culture dish (Corning, Lowell, MA, USA) and the organ of Corti was immersed in the DF12 + 10% FBS. Close attachment of the organ of Corti to the glass coverslip was established by aspirating DF12 + 10% FBS. The dish containing the cochlear explant was placed on the ice for half an hour, and then 1.5 mL of DF12 + 10% FBS was added to the dish. The organ of Corti explant was maintained at 37 °C in a 5% CO_2_ incubator with humidity.

### 3.4. Cisplatin and LBP Treatment

The organ of Corti explants were divided into four groups (*n* = 7 per treatment condition): (I) the organ of Corti explants cultured in DF12 + 10% FBS for 72 h as the control; (II) the organ of Corti explants cultured in control medium with LBP (150 μg/mL) for 24 h and subsequently exposed to cisplatin (20 μM) for 48 h; (III) the organ of Corti explants cultured in control medium with LBP (600 μg/mL) for 24 h and subsequently exposed to cisplatin (20 μM) for 48 h; (IV) the organ of Corti explants were cultured for an initial 24 h in control medium and then exposed to cisplatin (20 μM) for the next 48 h.

### 3.5. Assessment of Hair Cells Death

At the end of treatment, the specimens were fixed in 4% paraformaldehyde for 4 h in 0.1 M phosphate buffer (pH 7.4). Specimens were rinsed in 0.1 M PBS, incubated in 0.25% Triton X-100 for 5 min and immersed in FITC-labelled phalloidin (1:800; Sigma-Aldrich, St. Louis, MO, USA) in PBS for 30 min. Labeled hair cells were observed under a fluorescence microscopy(magnification 200×; NIKON, Tokyo, Japan). The number of hair cells was counted at each field from a single sample. Each group had seven samples. The average of hair cell counts from seven samples was evaluated.

### 3.6. ROS Levels in Organ of Corti Explant

ROS production in organ of Corti explant was assayed using a fluorescent dye 2′,7′-dichlorofluorescein diacetate (DCFH-DA) (Beyotime Biotech, Nantong, China) after treatment with cisplatin for 48 h. Briefly, the supernatant of the organ of Corti explant was removed, and the specimens were washed twice with cold 0.01M PBS (pH 7.4) and incubated with DCFH-DA (10 μmol/L) at 37 °C for 30 min. After DCFH-DA treatment, the chemical was removed and the specimens were washed three times with PBS. The fluorescence was read at 485 nm excitation and 530 nm emission under an inverted fluorescence microscope. Image Pro-Plus Version 6.0 software [31] was applied for measuring the fluorescence intensity of at least six fields per dish. Three parallel experiments were performed.

### 3.7. Measurement of Mitochondrial Membrane Potential (ΔΨ_m_)

Mitochondrial ΔΨ_m_ in hair cells was estimated using fluorescent probe JC-1, which exists in two forms: monomer and J-aggregate. JC-1, which has been widely used to assess the changes of ΔΨ_m_, is a lipophilic fluorescent cation that incorporates into the mitochondrial membrane, where it can shift from aggregates to monomer due to ΔΨ_m_ decrease. At low mitochondrial ΔΨ_m_, JC-1 exists mainly in a monomeric form which emits green fluorescence. At high mitochondrial ΔΨ_m_, this molecule forms aggregates which emit orange-red fluorescence. The dye in the healthy cells, in which mitochondrial ΔΨ_m_ is high, forms J-aggregates and emits orange-red fluorescence [32]. A break-down of mitochondrial ΔΨ_m_ is a marker of apoptosis [33,34], resulting in a decrease of orange-red fluorescence and increase of green fluorescence. JC-1 was excited with a 488-nm argon laser, and the shift from orange-red to green fluorescence indicated the decline of ΔΨ_m_.

After treatment with cisplatin for 48 h, the culture medium was removed, and the cultures were washed twice with cold 0.01M PBS (pH 7.4) and incubated with JC-1 (Beyotime Biotech, Nantong, China) (5.0 μg/mL) at 37 °C for 20 min. After incubation, the chemical was removed and the specimens were washed three times with PBS and placed in DF12. The fluorescence was read at 488 nm excitation and 530 nm emission for green, and at 540 excitation and 590 emission for orange-red. The ratios of orange-red/green JC-1 fluorescence intensity were calculated using an inverted fluorescence microscope. Typically, cells with a healthy population of mitochondria with a high mitochondrial ΔΨ_m_ have a high ratio of orange-red/green fluorescence intensity, whereas cells with declining mitochondrial ΔΨ_m_ have a low ratio of orange-red/green fluorescence intensity. Image Pro-Plus 6.0 software [31] was applied to for measuring the fluorescence intensity of at least six fields per dish. Three parallel experiments were performed.

### 3.8. Statistical Analysis

Fluorescence intensity for ROS and mitochondrial ΔΨ_m_ was measured using Image Pro-Plus 6.0. All statistical analyses were performed using stata version 10.0 software [35]. Data from at least three independent experiments were expressed as means ± SD. Comparisons of the means of different groups were made using one-way analysis of variance (ANOVA) or Kruskal-Wallis test. *P*-value below 0.05 was considered to be significant.

## 4. Conclusions

In conclusion, we show for the first time that LBP has a protective effect against cisplatin induced hair cell damage. The antioxidant ability of LBP plays a vital role in attenuating oxidative damage in hair cells induced by cisplatin. In addition, we demonstrate that the mechanism of the protective effect of LBP against cisplatin ototoxicity is partly attributed to the role of LBP in maintaining mitochondrial membrane potential.
